# Clinical Trials of Adult T-Cell Leukaemia/Lymphoma Treatment

**DOI:** 10.1155/2012/932175

**Published:** 2012-02-14

**Authors:** Ambroise Marçais, Felipe Suarez, David Sibon, Ali Bazarbachi, Olivier Hermine

**Affiliations:** ^1^Department of Hematology, Necker Hospital, 75473 Paris Cedex 15, France; ^2^Department of Internal Medicine, American University of Beirut, Beirut, Lebanon

## Abstract

Adult T-cell leukaemia/lymphoma (ATLL) is an aggressive malignancy of mature activated T cells caused by human T-cell lymphotropic virus type I (HTLV-1). Prognosis is severe because of intrinsic chemoresistance and severe immuosuppression. Four different subtypes are described with different outcomes, and treatment strategies vary according to the different clinical courses. Japanese trials show that combinations of chemotherapy can increase the response rates especially in the lymphoma subtype. However, patients have a high rate of relapse and the outcome remains extremely poor. Recently, a worldwide meta-analysis demonstrated that the combination of Zidovudine and Interferon-alpha (IFN) is effective in the leukemic subtypes (smoldering, chronic, and acute) and influences favorably the course of the disease. In order to prevent relapse, clinical trials testing new drugs such as monoclonal antibodies or combinations such as arsenic/IFN are needed. Finally, allogeneic stem cell transplantation is a feasible option but bears a very high rate of complications.

## 1. ATL Classification and Response Criteria

The classification first described by Shimoyama (1991) used for the initial staging distinguishes four subtypes, which differ regarding their presentation and outcome. This classification has been very useful for comparison between different studies [1]. 

The complex presentation with both leukemic and lymphomatous components makes response assessment difficult. Recently, an international consensus meeting established new response criteria [2].

Complete response (CR) is defined as the disappearance of all measurable tumor lesions (including normalization of lymph node size) and normalization of absolute lymphocyte (including flower cells less than 5%) count below 4 × 109/L. Unconfirmed CR is defined as a reduction of 75% of the tumor size and normalization of absolute lymphocyte (including flower cells) count below 4 × 10^9^/L. Partial response (PR) is defined as a reduction of 50% of tumor size and absolute lymphocyte count. Progressive disease is defined as an increase of 50% of the tumor size and/or absolute lymphocyte count. These response criteria require that each criterion is present for at least 4 weeks.

Treatment of ATL is usually dependent on the ATL subtype. Patients with aggressive forms (acute and lymphoma) have a very poor prognosis because of intrinsic chemoresistance, a large tumor burden, hypercalcemia, and/or frequent infectious complications due to profound immune deficiency. Multiple Japanese trials in aggressive ATL clearly demonstrated that although combinations of chemotherapy, in particular those designed for treatment of aggressive non-Hodgkin lymphomas or acute lymphoblastic leukemia, have improved the response rates particularly in ATL lymphoma, they failed to achieve a significant impact on long-term survival. Patients with indolent ATL (chronic or smoldering subtypes) have a better prognosis. However, recent Japanese data showed a poor long-term outcome when patients are managed with a watchful-waiting policy until progression and even worse when patients are treated upfront with chemotherapy [3].

## 2. Conventional Chemotherapy

The Japan Clinical Oncology Group (JCOG) has conducted six successive prospective clinical trials. All these trials are based on conventional chemotherapy, with various dose and administration modalities. The first trial JCOG 7801 used VEPA (a CHOP-like regimen that contained vincristine, cyclophosphamide, prednisolone, doxorubicin). The CR rate was only 17% with a median survival time of 5 months. The second trial, JCOG 8101, was a randomized phase III study, which included 54 patients and compared VEPA regimen with VEPA-M (VEPA plus methotrexate) [4]. Although the CR rate was improved in the VEPA-M group (37%), no differences in median survival time (7.5 months) and overall survival (8% at 4 years) were noted.

The third trial, JCOG 8701, was a phase II study with a more aggressive regimen (LSG 4), which combined 3 successive regimens: VEPA-B (VEPA plus bleomycin), M-VEPA (MTX, vindesine, cyclophosphamide, prednisolone, doxorubicin), and VEPP-B (vincristine, etoposide, procarbazine, prednisolone, and bleomycin). The CR rate was improved to 42%. However, median survival rate and overall survival were poor with a median survival time (MST) of 8 months and overall survival rate of 12% at 4 years. These trials enrolled also patients with other subtypes of NHL. MST was 44 months versus 8 months in the ATL group.

Following these initial trials, JCOG designed specific regimens targeting ATL. The JCOG9109 trial (a phase II study conducted between 1991 and 1993) used pentostatin-containing regimen but did not show any improvement (MST 7.4 months and 2 years overall survival rate: 15%) [5].

JCOG 9303 was conducted between 1994 and 1996 and used more intensive multiagent chemotherapy [6]. Treatment was designed as follows: VCAP (Vincristine, cyclophosphamide, doxorubicin, prednisolone), AMP (Doxorubicin, ranimustine, prednisolone), and VCEP (vindesine, etoposide, carboplatin, prednisolone) and include intrathecal injection of methotrexate and aracytine. The use of Granulocyte Colony Stimulating Factor (GCSF) was systematic. Results were encouraging with a CR rate of 35%, an MST of 13 months versus 8 months with historical control CHOP-like regimen. The 2-year OS was 31%. MCNU and carboplatin were used because their activity is not affected by the expression of P-glycoprotein, a product of MDR1, which is frequently expressed by ATLL cells.

In order to confirm these results, a phase III study (JCOG9801) was conducted between 1998 and 2003. This study compared two arms of treatment: VCAP-AMP-VECP versus biweekly CHOP. It included 118 patients (81 acute subtype and 26 lymphoma subtype) [7]. Response rate was higher in the experimental arm (40% versus 25%). Progression-free survival at 1 year was 28% versus 16%, and overall survival was 24% versus 13%. There was a statistically significant difference only in a subgroup analysis (patients younger than 56 years old, poor PS).

## 3. Allogeneic Stem Cell Transplantation

As most of patients relapse after conventional chemotherapy, allogeneic stem cell transplantation (alloSCT) seems to be an attractive option as consolidation treatment. Most of the reports come from Japan. A number of retrospective studies have confirmed that alloSCT uses either myeloablative conditioning (MAC) or reduced-intensity conditioning (RIC) as a feasible treatment option for ATL patients. The largest retrospective study has been reported recently [8]. This study includes 386 patients allografted between 1995 and 2005. After a median followup of 41 months, 3-year overall survival was 33%. Among patients who received related transplants, donor HTLV-I seropositivity adversely affected disease-associated mortality. Recently, the long-term results of a series of 30 patients who received an RIC was reported. Overall survival rate and progression-free survival rates were 36% (95% IC, 21 to 25%) and 31% (95% IC, 17 to 45%), respectively, [9]. However, the number of ATL patients eligible for alloSCT is very limited because of the low CR rate especially in the acute form, poor performance status, severe immunosuppression, age at disease (median age at onset: 60 years old), and low probability of finding suitable donors in patients from ethnic minorities.

## 4. Alpha Interferon (Zidovudine) AZT

Even if this treatment association is frequently referred to “antiviral therapy”, mechanism of action is not fully understood yet. The combination of Zidovudine (AZT) and alpha interferon (IFN) was first reported in 2 phase II studies [10–12]. High response rate was observed particularly in previously untreated acute ATL. The efficacy of this combination was confirmed in a French trial using AZT/IFN in 19 newly diagnosed ATL patients, and in a UK clinical trial using AZT/IFN in 15 ATL patients [13, 14]. In a recent prospective Phase II study in the USA, 19 ATL patients received infusional chemotherapy (EPOCH regimen) until maximal response, followed by antiviral therapy with daily AZT, lamivudine, and IFN. However, because of disease progression, only 6 patients received antiviral therapy [15].

A worldwide meta-analysis was recently performed on ATL survival since 1995 [16]. In this study, different treatment strategies for ATL has been compared, namely, antiviral therapy alone, chemotherapy alone, and chemotherapy followed by maintenance antiviral therapy in 254 ATL patients treated in the USA, the UK, Martinique, and continental France (116 acute ATL, 18 chronic ATL, 11 smoldering ATL, and 100 ATL lymphoma). Five-year OS rates were 46% for 75 patients who received first-line antiviral therapy, 20% for 77 patients who received first-line chemotherapy, and 12% for 55 patients who received first-line chemotherapy followed by antiviral therapy.

Patients with leukemic forms significantly benefited from first-line antiviral therapy, whereas patients with ATL lymphoma had a better outcome with chemotherapy. In acute ATL, first-line antiviral therapy alone resulted in a significant survival advantage (5-year OS of 28%) as compared with first-line chemotherapy with or without maintenance antiviral therapy (5-year OS of 10%). Achievement of CR with antiviral therapy resulted in 82% 5-year survival. In chronic and smoldering ATL, antiviral therapy resulted in 100% 5-year survival. In ATL lymphoma, first-line antiviral therapy resulted in a significant survival disadvantage (median and 5-year OS of 7 months and 0%, resp.) compared with first-line chemotherapy with or without maintenance antiviral therapy (median and 5-year OS of 16 months and 18%, resp.). Finally, a multivariate analysis confirmed that first-line antiviral therapy significantly improves overall survival of ATL patients (HR 0.47; 95% CI 0.27–0.83; *P* = 0.021).

## 5. Arsenic Trioxide (AsO_3_)

Arsenic trioxide synergizes with IFN to induce cell cycle arrest and apoptosis in HTLV-I infected and fresh ATL cells through rapid shut-off of the NF-*κ*B pathway and a delayed shut-off of cell cycle-associated genes, secondary to Tax degradation by the proteasome [17–19]. Although it has been demonstrated that arsenic and IFN cooperate to cure murine ATL derived from *Tax *transgenics through selective eradication of leukemia-initiating cell (LIC) activity. This strongly suggests that LIC activity is dependent on continuous Tax oncogene expression. Hence, addition of arsenic to AZT/IFN, through elimination of LIC activity, may result in long-term disease eradication and potential cure [20]. A recent prospective phase II study evaluated the efficacy and safety of the combination of arsenic, IFN, and AZT in 10 newly diagnosed chronic ATL patients. The response rate was 100% including 7 CR, 2 CR but with more than 5% circulating atypical lymphocytes, and 1 partial response. Side effects were moderate and mostly hematologic [21]. We have also recently reported a series of 11 patients with ATL (3 lymphoma type, 3 chronic, and 5 acute) treated with arsenic/IFN after induction chemotherapy [22]. At initiation of AsO_3_, 4 patients were in CR, 2 in PR, and 5 in progression. 10 patients received AsO_3_ during 3 to 8 weeks. One progressed 3 days after starting AsO_3_ and 6 patients died. All were progressive at time of AsO_3_ initiation. 5 patients survived: 3-lymphoma type in CR (25, 31, 46 months of followup), 1 acute in CR (9 months followup), and 1 chronic in PR (39 months followup). Tolerance was acceptable with peripheral neuropathy (*n* = 4), hand and foot syndrome (*n* = 3), and drug eruption (*n* = 3, including 2 toxic epidermolysis). While preliminary, these observations nevertheless suggest that in ATL patients arsenic/IFN efficiently targets ATL LIC activity and may be useful as a consolidation therapy for those patients achieving a satisfactory response to induction therapy.

## 6. Specific Monoclonal Antibodies

ATL cells express CD25 (alpha-chain of IL2 receptor). A first trial reported use of antiCD25 antibody on 19 patients. Authors obtained 6 responses (two CR, four PR) that lasted from 9 weeks to more than 3 years [23].

A second study used CD25 coupled with YTRIUM-90. Seven of 18 patients treated (one with chronic ATL and 6 with acute ATL) obtained a partial remission. The duration of these partial remissions ranged from 1.6 to 22.4 months (mean, 9.2 months). Two patients achieved CR. One died 36 months after initiation of therapy from a secondary AML and the other patient was still in CR at time of publication [24].

A neutralizing monoclonal antibody to the transferrin receptor (mAb A24) has been designed and induces apoptosis of ATLL cell lines and primary ATL cells [25]. Thus far, only preclinical studies have been performed (Hermine et al., personal communication).

## 7. Anti-CC Chemokine Receptor 4 (CCR4)

ATL cells express the CC chemokine receptor 4 (CCR4). KW-0761 is a defucosylated humanized antibody with enhanced antibody-dependent cellular cytotoxicity (ADCC) that binds CCR4. A phase I study reports 13 patients with CCR4-positive relapsed ATL treated with KW-0761. Overall response rate (ORR) was 31%: 2 CRs and 2 PRs [26]. A pivotal phase II study has been recently presented on the 15th International Conference on Human retrovirology HTLV and related viruses. The primary end point was ORR. Twenty-eight patients with relapsed ATL were enrolled. Among the 26 pts evaluable for efficacy, the ORR was 50% with 8 CRs and 5 PRs with response rates in each affected lesion being 100% (13/13) for peripheral blood, 63% (5/8) for skin, and 25% (3/12) for lymph node disease, respectively. The treatment schedule was one weekly perfusion (1.0 mg/kg) for 8 weeks. Adverse events were mild to moderate.

## 8. Watch-and-Wait Policy

Patients with smoldering or chronic ATLL subtype have a better prognosis than patients with aggressive forms (acute and lymphoma) and have been considered as indolent forms. Many patients have been managed with a watch-and-wait policy until disease progression or treated with chemotherapy when poor prognostic factors were present. A recent published Japanese study reported 90 patients with indolent form (65 chronic and 25 smoldering) [3]. Forty-four (49%) patients progressed to aggressive form with a median time of transformation of 18.8 months (range 0.3 months to 17.6 years) and 41 died. Median survival time was 4.1 year. No difference between the two subtypes (chronic and smoldering) was observed. The estimated 10-year survival rate was 25, 4% (95% CI, 15.3–36.8%). This study shows that even, in the indolent subtype, prognosis is poor. Moreover, patients who received chemotherapy had a worse prognosis and a shorter life expectancy than patients who were treated was followed with watchful waiting. These results underscore the need for further improvement in the treatment of patients with otherwise indolent forms of ATL.

## 9. Agents That Have Shown Efficacy on T-Cell Lymphoma outside HTLV-1 Infection

 Currently, it is not yet clear whether or not T-cell lymphoproliferation associated with HTLV-1 infection is, with respect to oncogenic mechanisms, different from other T-cell lymphoma and as such whether or not they may benefit from drugs approved or in the development in T-cell lymphoma. We discuss the potential benefit of five agents currently developed in the treatment of T-cell lymphomas: agents with potential cytotoxic effect (pralatrexate and Bendamustine), T-cell-targeted immunotherapy (Alemtuzumab) and agents interacting with major cellular signaling pathways and/or viral homeostasis (Histone deacetylase inhibitors, Lenalidomide).

### 9.1. AntiFolate (Pralatrexate)

Pralatrexate is a new antifolate that was designed to be efficiently internalized by the reduced folate carrier (RFC). A prospective study has shown its relative efficacy on 111 patients with relapse T-cell lymphoma [27]. Major lymphoma subtypes were peripheral T-cell lymphoma (PTCL) and angio-immunoblastic T-lymphoma (AITL). Only one patient in this study had an ATL. The response rate in 109 evaluable patients was 29% (32 of 109), including 12 complete responses (11%) and 20 partial responses (18%), with a median duration of response of 10.1 months. Median PFS and OS were, respectively, 3.5 and 14.5 months. The U.S. FDA approved Pralatrexate for cutaneous T-cell lymphoma (CTCL) in 2009.

### 9.2. Histone Deacetylase Inhibitors (Vorinostat, Romidepsin, Panobinostat, and Belinostat)

Histone deacetylase inhibitors HDAC inhibitors (HDACI) are a new class of drugs whose activity was initially designed on transcriptional activity by acting on chromatin epigenetic modification, histone deacetylation. However, their antitumor activity seems to occur through others pathways. Indeed, it has been shown that they also increase acetylation of other proteins such as nuclear transcription factors. Whatever the mechanism of action, exposure of cancer cells to HDAC inhibitors results in growth arrest, cellular differentiation, and apoptosis.

Two of these agents (vorinostat and romidepsin) have been approved in the USA for the treatment of relapsed and refractory CTCL. In these studies, they have been used as a single agent. Studies are ongoing to evaluate their efficacy on PTCL.

Vorinostat was evaluated in a phase II study. This study included 74 pts with CTCL who had failed at least two prior systemic therapies [28]. The primary end point was overall response rate (ORR). ORR was 29.7%. Median time to objective response was 56 days (range, 28–171). Median duration of response was not reached but estimated to be more than 185 days (range, 34–441). Major side effects were diarrhea (49%), fatigue (46%), nausea (43%), and anorexia (26%). Eleven patients required dose modification and nine discontinued due to adverse event. On the basis of this study, the U.S. FDA approved Vorinostat for CTCL in October of 2006.

Romidepsin was the second HDAC inhibitor that was approved by the U.S. FDA for CTCL in 2009. Two phase 2 trials were conducted in patients with CTCL with the primary goal of determining response rate and tolerance toxicity profile. The first trial included 71, and 96 patients were treated on a second trial. Response rates were 34% for both studies with median durations of 13.7 and 15.4 months, respectively, [29]. Side effects that were acceptable included nausea, vomiting, fatigue, and transient thrombocytopenia and granulocytopenia. Romidepsin was approved for CTCL after these two studies.

Recently, romidepsin was evaluated on PTCL. A phase 2 study reported forty-seven patients with PTCL of various subtypes including PTCL not otherwise specified (NOS), AITL, ALK-negative anaplastic large cell lymphoma, and enteropathy-associated T-cell lymphoma [30]. All patients received prior systemic therapies. Eighteen (38%) received stem cell transplantation. Overall response rate was 38% (95% confidence interval 24%–53%) with 8 CR and 9 PR. The median duration of overall response was 8.9 months (range, 2–74). Moreover, 6 responses were observed among the 18 patients with prior alloSCT. Side effects were acceptable.

To our knowledge, these drugs have not been yet evaluated in ATL as a single therapy or in combination with other drugs in induction therapy. However, Ramos et al. have reported a clinical trial using IFN-AZT with valproic acid (HDAC inhibitor) during the maintenance treatment phase [31]. The authors hypothesized that HDAC inhibitors could reactivate latent HTLV-1 in ATLL cells harboring intact provirus and help eliminate residual disease. Thirteen patients were enrolled. One showed a serial decrease in clonal ATLL disease followed by PCR. Using fresh cells from this patient treated *ex vivo* with Vorinostat, the authors showed an increase of HTLV-1 expression and an induction of cell death. However, in this study, induction of a putative immune response against virus-infected cells was not addressed.

### 9.3. Monoclonal Antibody

Alemtuzumab (CAMPATH-1H) is an anti-CD52 antibody that is approved for chronic lymphoid leukemia treatment. It has been showed that it is effective on T-cell prolymphocytic leukemia with high response rate in a prospective study including 39 patients with T-PLL treated with CAMPATH-1H [32]. The overall response rate was 76% with 60% CR and 16% partial remission (PR). These responses were durable with a median disease-free interval of 7 months (range, 4–45 months). In ATL, experience is limited to case report [33]. In addition, a recent study reported efficacy of the association of alemtuzumab and pentostatin in various types of PTCL including one case of ATL, which was in CR [34]. However, association Campath with conventional chemotherapy in PTCL has shown relative efficacy but high rate of infections.

### 9.4. Lenalidomide

Lenalidomide is a drug currently used for myeloma treatment. Studies have reported its use as single therapy for PTCL treatment. An interim report for a phase 2 clinical trial has been reported [35]. Patients with recurrent and refractory T-cell lymphomas other than mycosis fungoides and untreated patients ineligible for combination chemotherapy were prescribed oral lenalidomide (25 mg daily) on Days 1 to 21 of each 28-day cycle until disease progression. At the time of this interim analysis, 24 patients were enrolled in this study, and 23 were evaluable for response. The overall response rate was 7 (30%) of 23; all were in partial responses. Two patients had stable disease for ≥5 cycles. Median PFS was 96 days (range, 8–696 days). Median OS was 241 days (range, 8–696 days). The most common grade 4 adverse event was thrombocytopenia (33%).

### 9.5. Bendamustine

Bendamustine is a cytotoxic agent that has been recently approved for the treatment of CLL and indolent lymphoma such as follicular lymphoma. This drug shows structural similarities with alkylating agents or antimetabolites. A phase II study tests his efficacy on relapsed or refractory peripheral T-cell lymphoma. The primary end point is ORR (CR, CRu, PR). Preliminary results have been shown recently for the first 38 patients (G. Damaj et al. 11th International Conference on Malignant Lymphoma, abstract n°126). ORR was 47%: CR + CRu in 11 patients (29%), PR in 7 patients (18%). 20 patients experienced relapse. At the time of analysis, the median duration time for responder patients was 157 days (range, 14–350). The most adverse events (grade 3 and 4) were neutropenia and thrombopenia.

## 10. Strategy

**Figure 1:**
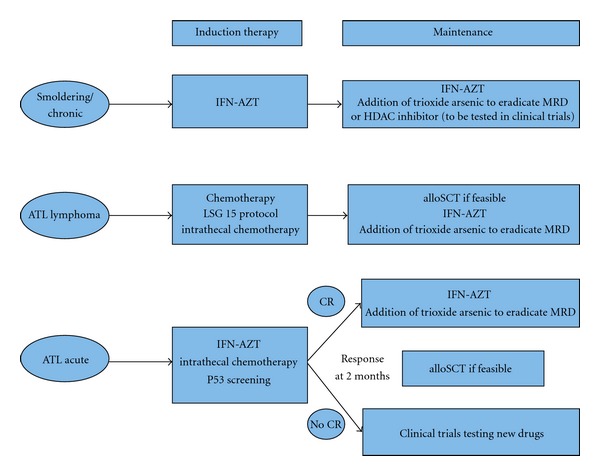
Recommended treatment strategy for patients with acute, lymphoma, or chronic/smoldering ATL (CR: complete remission; MRD: minimal residual disease; AZT: zidovudine; IFN: interferon-alpha; alloSCT: allogeneic stem cell transplantation).

Suggested treatment strategies according to clinical presentation are described in Figure 1.

### 10.1. Chronic and Smoldering ATL

Patients with chronic and smoldering ATL have a better prognosis compared to patients with aggressive forms (acute and lymphoma). However, as it has been shown in a recent Japanese study, long-term survival is dismal when these patients are managed with a watchful-waiting policy until disease progression. Moreover, patients who received chemotherapy alone had a poorer outcome indicating that this may be detrimental in these subtypes [3]. So far, no clear prognostic factors have been yet defined in order to predict transformation to an aggressive form and treated patient who are at risk.

Our point of view is that most of patients with chronic and smoldering ATL should be treated. In the recent worldwide meta-analysis, patients with chronic/smoldering ATL who received first-line therapy by AZT-IFN only had an excellent survival (100% OS beyond 5 years). Thus, outside the context of clinical trials, the current standard therapy of chronic and smoldering ATL is combination therapy with AZT and IFN. This requires, however, continuous therapy. Treatment should not be interrupted as relapse always occurs when treatment is stopped. The recommended starting dose is AZT 600 to 900 mg/day (in 3 divided doses) and interferon-alpha (5 to 6 million IU/m2/day). Usually, after one month, AZT dose can be titrated down to 600 mg/day in 2 divided doses and IFN dose can be reduced to 3 to 5 million IU/day or alternatively 1.5 *μ*g/kg of pegylated IFN weekly. Based on preclinical studies, clinical trials are testing the effect of adding arsenic to the AZT/IFN combination as a consolidation therapy with the aim of then stopping therapy and achieving cure by potential elimination of leukaemia-intiating cells [17–20].

### 10.2. ATL Lymphoma

As has been shown in the recent meta-analysis, the combination AZT-IFN is less effective than first-line chemotherapy in ATL lymphoma [16]. Therefore, chemotherapy should be the preferred option. However, recent unpublished results from the UK suggest that combination of antiviral therapy with CHOP chemotherapy is superior to CHOP alone in patients with ATL lymphoma [36]. Use of chemotherapy is based on the Japanese experience across different trials. The LSG15 protocol is the “standard of care.” It is based on multiple drugs. When treated with this LSG15 protocol, ATL lymphoma patients achieved a better CR rate (66.7%) than acute type (19.6%) or chronic type (40.0%). However, relapse occurs rapidly and overall survival rate is low [6]. Therefore, a consolidation therapy is critical. Whenever possible, allogeneic SCT should be considered [8]. For patient failing to achieve remission after chemotherapy or lacking a suitable donor, a consolidation strategy should be discussed. Based on preclinical data, ongoing clinical trials are testing the efficacy of two cycles of arsenic/IFN maintenance as a consolidation procedure following achievement of CR with encouraging preliminary results [22]. Moreover, the addition of AZT/IFN or other novel therapies to chemotherapy may help to achieve remission. HDAC inhibitor might be tested in this indication to induce an immune response against residual tumor cells.

### 10.3. Acute ATL

Combination chemotherapy regimens have little effect in acute ATL. Even if the most intensive regimen (LSG-15) have increased response rate, MST and OS are low [6, 7]. In the recently published meta-analysis on antiviral therapy for ATL, treatment of acute ATL patients with AZT and IFN showed a higher response rate and significantly prolonged survival. Moreover, patients who achieved CR had a long-term response [16]. Outside the context of clinical trials, the current standard therapy of acute ATL is combination therapy with AZT and IFN. However, it can be difficult to manage patients presenting with bulky tumor or severe hypercalcemia not responding to bisphopshonates, and initial chemotherapy is sometimes required. It would be helpful to predict which patients in the acute form will benefit from this approach. Preliminary results indicate that patients with wild-type functional p53 are more likely to respond to AZT/IFN combination [37]. We, therefore, recommend evaluating p53 by a functional assay in all patients while the treatment is initiated [38]. Long-term disease control requires, however, continuous therapy, since relapse is always noted when treatment is stopped. The recommended dose is the same as with chronic/smoldering form. As in lymphoma subtype, allogeneic HSCT should be considered for young patients with acute ATL and a suitable donor [38]. As in other ATL subtype, based on preclinical data, ongoing clinical trials are testing the efficacy of arsenic/IFN maintenance following achievement of CR.

### 10.4. Supportive Therapy in ATL

Hypercalcaemia associated with aggressive ATL should be managed with treatment of the disease, hydration, and bisphosphonate therapy. Trimethoprim-sulfamethoxazole, valacyclovir, and antifungal agents are recommended for the prophylaxis of *Pneumocystis jiroveci* pneumonia, herpes simplex virus, and fungal infections, respectively; in the Japanese Trials, prophylaxis with antistrongyloides agents, such as ivermectin or albendazole, should be considered in order to avoid systemic infection in patients with a history of past and/or present exposure to the parasite. Intrathecal prophylaxis should be considered for patients with aggressive ATL even in the absence of clinical symptoms because more than half of relapses at new site after chemotherapy occur in the central nervous system.

## 11. Conclusion

The combination of AZT and IFN is highly effective in the leukemic subtypes of ATL and should be considered as standard in first-line therapy in that setting. This combination has clearly changed the natural history of the disease through achievement of a significantly improved long-term survival in patients with smoldering and chronic ATL as well as a subset of patients with acute ATL. Prior exposure to chemotherapy increases the rate of complications and of acquiring p53 mutations. We, therefore, recommend that the combination of AZT and IFN is used as a first-line treatment in the leukemic forms and that treatment is initiated with high doses of both agents since reduced doses are often not effective. ATL lymphoma patients benefit from initial induction therapy based on aggressive chemotherapy regimen but constantly relapse and have a poor prognosis. Addition of AZT-IFN in combination with chemotherapy may increase response rate but its long-term effect remains to be determined. We recommend, for those in whom alloSCT is not feasible, that a consolidation treatment with AsO_3_ is considered, followed by maintenance therapy with AZT/IFN. This approach should be tested in future clinical trials. Prophylaxis of opportunistic infections and supportive therapy are mandatory. In order to prevent the occurrence of resistance and relapse, clinical trials assessing additional targeted therapies such as arsenic/IFN combination or monoclonal antibodies, particularly the promising anti-CCR4 antibodies, are mandatory after achieving CR. Finally, allogeneic SCT should be considered in suitable patients. HDAC inhibitor may be also an interesting option. Currently, due to the poor outcome of patients with aggressive ATL (acute and lymphoma forms), phase II studies are mandatory in the near future. In chronic form, it is time to set up phase III studies to assess new drugs to avoid relapse for patients treated with AZT-IFN.
